# LC-MS based cell metabolic profiling of tumor cells: a new predictive method for research on the mechanism of action of anticancer candidates†

**DOI:** 10.1039/c8ra00242h

**Published:** 2018-05-08

**Authors:** Hua Wang, Jia-hui Hu, Cui-chai Liu, Min Liu, Zheng Liu, Li-xin Sun

**Affiliations:** Department of Pharmaceutical Analysis, School of Pharmacy, Shenyang Pharmaceutical University Shenyang 110016 China sunlixin67@yahoo.com +86 02443520600; GLP Center, School of Life Science and Biopharmaceutics, Shenyang Pharmaceutical University Shenyang China

## Abstract

In the process of anticancer drug development, research on the mechanism of action remains a major obstacle. In the present study, a cell metabolic profiling based discriminatory model was designed to give general direction on anticancer candidate mechanisms. Firstly, ultra-performance liquid chromatography in tandem with high-definition mass spectrometry was applied to obtain a comprehensive metabolic view of 12 human tumor cells. Secondly, multivariate data analysis was used to assess the metabolites’ variations, and 42 metabolites were identified as the main contributors to the discrimination of different groups. Then a metabolite-based prediction model was constructed for the first time and verified by cross validation (*R*^2^ = 0.909 and *Q*^2^ = 0.869) and a permutation test (*R*^2^ = 0.0871 and *Q*^2^ = −0.4360). To validate if the model can be applied for mechanism prediction, 4 independent sample sets were used to train the model and the data dots of different drugs were located in different regions. Finally, the model was applied to predict the anticancer mechanism of two natural compounds and the results were consistent with several other studies. Overall, this is the first experimental evidence which reveals that a metabolic profiling based prediction model has good performance in anticancer mechanism research, and thus it may be a new method for rapid mechanism screening.

## Abstract 2

The comprehensive workflow of construction and evaluation of a predictive model based on cell metabolic profiling.

## Graphical abstract

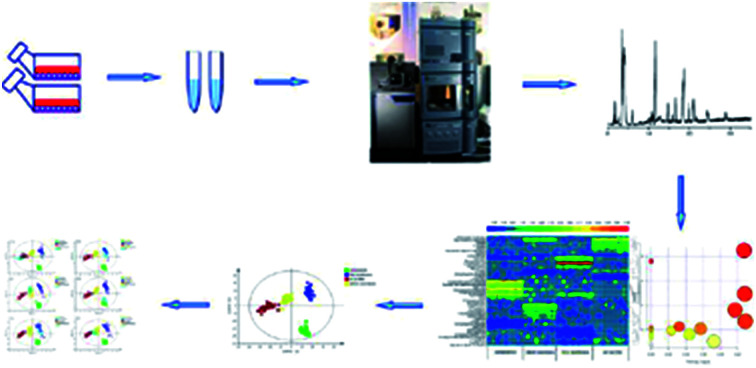

## 1. Instruction

Progress in early detection and treatment, especially in the research of anticancer drugs, has been gradually changing cancer from a guaranteed death sentence into a chronic illness.^1^ The research and development of anticancer drugs is a complicated process including drug discovery, preclinical tests, and clinical trials. In this complex process, research on mechanism of action is of great significance to an anticancer candidate being developed and reaching the market.^2^ The research on mechanism of action mainly focuses on revealing the main targets of candidates and the signalling pathways associated with effects, discovering potential side effects, studying drug resistant mechanisms and instructing rational drug design.^3^ However, research on the mechanism of anticancer drug action is a very costly and time-consuming step, and it is reported that nearly one-third of the capital lost on all drug failures, $8 billion, is attributed to the failure of drug pharmacology and toxicology.^4^ Therefore, more-robust and faster approaches for predicting the preliminary mechanism of action of anticancer candidates are urgently needed to point out directions for further studies and improve the efficacy of drug development.

In a previous study, on the basis of nucleotide content, a discriminatory model was developed which was capable of distinguishing different types of anti-tumor agents and preliminarily predicting the possible mechanism of compounds to a certain extent.^5,6^ However, the model was not satisfactory, and its prediction accuracy and stability were not perfect. This result could be attributable to the limitation of the method. The previous model only investigated the changes in intracellular nucleotide content. Actually, because of the extensive interactions and biochemical consequences, most intracellular metabolites will be affected after treatment. To improve the reliability of mechanism predictions, it could be better to extend the database to cover as many intracellular metabolites as possible. Hence, a more global and powerful approach to study the correlation between changes in metabolites and mechanisms of action may be a novel method to predict the mechanism of anticancer candidates.

As an emerging discipline, cell metabolic profiling may provide full insight into integral perturbed metabolic profiles and help understand the effect caused by treatment. Thus it has been widely performed to study the mechanisms of antitumor active components with unclear modes. For instance, based on cell metabolic profiling Gao established an approach to investigate the antitumor action mechanism of a novel flavonoid derivate on HepG2 cells.^7^ Hu analyzed the metabolic changes induced by celastrol treatment in human cervical cancer cells and provided new evidence of the pharmacological mechanism of celastrol.^8^ Furthermore, some metabolic profile based discrimination models have been reported in evaluating clinical therapy, diagnosing disease,^9^ revealing dose effects,^10^ determining disease stage,^11^ and improving avian productivity.^12^ For example, a study has demonstrated the novel strategy that the metabolic profiling approach may be applicable to show the discrimination of metabolites between treated and control groups in human endothelial cells, and thus contribute to the assessment of the clinical therapeutic effect.^13^ However, no information is available on metabolic profiling applied to anticancer mechanism prediction. Herein, a metabolic profile, for the first time, was adopted to create an anticancer mechanism prediction model which might be beneficial for understanding the wide-ranging molecular interactions induced by agent treatment in tumor cells.

In this protocol, ultra-performance liquid chromatography in tandem with high-definition mass spectrometry was chosen as a sensitive analytical technique to reveal the biological information of different tumor cell lines. Then the metabolism changes were analyzed after incubation with clinical chemotherapeutic drugs which exhibit one of four different kinds of action mechanism, including antimetabolic agents (5-fluorouracil, methotrexate and cytarabine), agents which directly act on DNA (mitomycin C, cisplatin and etoposide), agents which affect microtubule dynamics (taxol, vincristine and docetaxel), and RNA interference agents (daunorubicin, epirubicin and dactinomycin D). With multivariate statistical analysis, drug-correlated key markers were described, and based on the metabolites, a generalizable orthogonal partial least squares discriminant analysis (OPLS-DA) prediction model was obtained for the first time. By analyzing the following new data (gemcitabine, carmustine, mitoxantrone, vindesine) that belong to the four categories previously mentioned, the prediction accuracy of the model was further validated. Finally, using the obtained model we successfully predicted the anticancer mechanism of two natural compounds (apigenin, diosgenin) that had exhibited cytotoxicity. Our study demonstrated for the first time that the cell metabolic profiling based novel prediction model may be a reliable method for preliminary mechanism research of anticancer candidates.

## 2. Material and methods

### 2.1 Chemicals and reagents

High-performance liquid chromatography (HPLC) grade methanol and acetonitrile were purchased from Fisher Scientific (Waltham, USA). Internal standard l-methionine sulfone and testosterone propionate were obtained from Shanghai Aladdin Biochemical Technology Co., Ltd. Dulbecco’s modified Eagle’s cell culture media (DMEM), Roswell Park Memorial Institute (RPMI) 1640 media, fetal calf serum (FCS) and 4-(2-hydroxyethyl)-1-piperazineethanesulfonic acid (HEPES) were all obtained from Gibco-BRL (Grand Island, New York, USA).

### 2.2 Cell lines and cell culture

All cell lines were obtained from the American Type Culture Collection (ATCC), (http://www.atcc.org/). Human pulmonary carcinoma cells (A549), human rhabdomyosarcoma cells (A204), human hepatocellular carcinoma cell lines (HepG2), human ovarian carcinoma cells (SKOV3), human breast carcinoma cells (T47D), and human breast adenocarcinoma cells (MCF7) were cultured in DMEM medium containing 3.7 g L^−1^ NaHCO_3_, and 10% FCS. Human fibrosarcoma cells (HT1080), human prostatic cancer cells (DU145), human prostate carcinoma cells (PC3), Henrietta Lacks strain of cancer cells (HeLa), human mouth epidermal carcinoma cells (KB), and human gastric carcinoma cells (SGC7901) were maintained in RPMI-1640 medium supplemented with 2.4 g L^−1^ HEPES, 2.0 g L^−1^ NaHCO_3_, and 10% FCS. The twelve tumor cell lines were incubated at 37 °C with 5% CO_2_ in a humidified air atmosphere for 4 or 5 days, with the culture duration selected to maintain cells under exponential growth and to reach 80% confluence.

### 2.3 Cell treatment and quenching

Cells were seeded at a density of 1.0 × 10^6^ cells into each T25 flask and cultivated for 24 h. According to the IC_50_,^14^ drugs were diluted with culture medium and added to flasks of treatment groups. After treatment with or without drugs for 48 h, the culture medium was removed from the flask and the cells were quickly washed twice with 3 mL of ice-cold phosphate-buffered saline (PBS, pH 7.4). The cells were rapidly quenched by liquid nitrogen (LN_2_), then suspended in ice-cold PBS by cell scraping with a pre-chilled cell scraper, which was performed on ice. The quenched biomass was then centrifuged (3 min, 1500 rpm) at 4 °C, with cell pellets collected and stored at −80 °C until further use. Each condition was applied to four replicates, three of which were applied for metabolic profiling and one for cell number counting to normalize the data. Cell counts were processed according to these steps: separation of the medium, then washing with cold PBS twice, detachment from flasks using 0.25% trypsin, and the final cell number was obtained using a hemocytometer.^15^

### 2.4 Sample preparation

The sample preparation followed a modified protocol.^16^ Briefly, to extract lipophilic metabolites, 300 μL of dichloromethane and 20 μL of internal standard (50 μg mL^−1^ methanol solution of testosterone propionate) were used and vortexed for 3 min. Maximum lysis of cell compartments was achieved by ultrasonic treatment for 1 min, allowing release of intracellular metabolites. Then the mixture was placed in an ice-water bath for 20 min, followed by a centrifugation at 12 000 rpm for 10 min at 4 °C. The supernatant was transferred to a new Eppendorf tube and evaporated to dryness under a gentle nitrogen stream at 40 °C. To extract the hydrophilic metabolites, 300 μL of methanol and 20 μL of internal standard (50 μg mL^−1^ methionine sulfoxide solution) were added to the residue. After vigorously vortexing for 3 min the mixture was placed in an ice-water bath for 20 min and centrifuged at 12 000 rpm for 10 min at 4 °C. The supernatant was collected and dried with a stream of nitrogen. Following this, the samples were reconstituted in 100 μL of acetonitrile–water (1 : 1,v/v) for analysis.

### 2.5 LC-MS analysis

The samples were analysed by an ACQUITY UPLC system coupled to a SYNAPT-G2-Si high-definition mass spectrometer from Waters (Milford, USA). The HILIC method helped to separate highly polar metabolites whereas the traditional RPLC method provided efficient separation of nonpolar metabolites. These two methods were complementary and together, can provide enhanced coverage of metabolites.

#### 2.5.1 RP-UPLC-MS analysis

RP-UPLC-MS was performed on a Phenomenex Kinetex XB C18 column (50 mm × 2.1 mm, 1.7 μm) at 40 °C with gradient elution. Mobile phases A and B were water containing 0.1% formic acid solution, and acetonitrile with 0.1% formic acid, respectively. The following gradient was used at a flow rate of 0.2 mL min^−1^: min 0, 15% B; min 6, 25% B; min 20, 50% B; min 40, 90% B; min 45, 15% B, terminating the run at 60 min. A re-equilibration at 15% B was maintained for 10 min before the next injection.

#### 2.5.2 HILIC-UPLC-MS analysis

HILIC-UPLC-MS analysis was performed on an ACQUITY BEH Amide column (100 mm × 2.1 mm, 1.7 μm) at 30 °C. Mobile phases A and B were 0.1% formic acid solution, and acetonitrile with 0.1% formic acid, respectively, and were delivered at 0.2 mL min^−1^. The gradient elution program started at 95% B (0–5 min) changing to 85% B (5–5 min), which was maintained for 10 min, and finally to 60% B (25–35 min) before returning to the starting conditions, followed by re-equilibration for 20 min.

For both RP and HILIC modes, the injection volume was 5 μL. The mass spectrometry was performed in electrospray positive ionization mode with mass ranges of 124–1000 Da and 8–1000 Da. Instrument settings were set as follows: ion voltage 3.0 kV, source temperature 120 °C, desolvation temperature 350 °C, cone voltage at 30 V and 20 V respectively, the cone gas flow 50 L h^−1^ and desolvation gas flow 600 L h^−1^. The scan time was set at 0.2 s, the interscan delay at 0.1 s. The same parameters were applied for the simultaneous MS and MS/MS fragments analyses, with a collision energy ramp from 10 to 35 eV.

### 2.6 Method validation

In order to investigate the repeatability of the method, we prepared six SGC cell samples in parallel, and seven steady peaks including internal standard were then chosen to calculate the RSD values using retention time and relative peak area. In addition, one identical sample was stored at room temperature after preparation and analyzed at 0, 2, 4, 8, 12 and 24 h, to validate the stability. On the other hand, to check the stability of the LC-MS system while running samples, quality control (QC) samples were injected at the beginning of the run and after every six real samples. Prior to analysis, QC samples were prepared by mixing equal aliquots of all of the samples in the identical corresponding dataset.^17^

### 2.7 Statistical analysis

All of the raw data were imported to the MarkerLynx 4.1 software (Waters) for peak alignment, background noise subtraction, and data reduction to obtain a metabolite features list containing aligned retention time and *m*/*z* values. The main parameters were set as follows: mass range 80–1000 Da, mass window 0.05 Da, minimum intensity 5% of base peak intensity, noise elimination level 6 and RT tolerance 0.5 min. To acquire consistent variables, the data was filtered further by removing any peaks with missing values (ion intensity = 0) greater than 20% in all samples.^18^ Finally, the data were log 10-transformed and Paretoscaled before the statistical analysis.

Raw data quality was checked by principal component analysis (PCA), an unsupervised method, with the SIMCA13.0 software (Umetrics AB, Umea, Sweden), both for each batch and for all batches together. Then a generic and powerful processing method known as OPLS-DA, which is able to effectively remove the non-correlated variations from *X*, was executed to identify the metabolic variations caused by treatments. Differential metabolites responsible for discrimination between the groups were selected on the basis of a threshold of variable importance in projection value from the OPLS-DA model. In parallel, one-way analysis of variance (ANOVA) along with a Bonferroni correction was applied to calculate the statistical difference of each variable whose VIP value was higher than 1.0, setting the significance level to 0.05 as a criterion with the SPSS 19.0 software. Heat map analysis was performed by MultiExperiment Viewer (version 4.8.1).

The receiver operating characteristic (ROC) curve was used to evaluate whether the differential metabolites were the independent factors of drug action. The discriminatory power of each candidate was ranked using the area under the curve (AUC). Biomarkers with AUC >0.50 were regarded as being highly sensitive and specific.

### 2.8 Metabolite identification

The putative identification of these discriminative metabolites was based on accurate mass, retention time, and MS/MS information. The levels of chemical identification were in accordance with the published guidelines for metabolomics studies.^19,20^ Level 1 was used for the direct matching of orthogonal data, such as the retention time and mass spectrum (MS and MS/MS spectra) using standard molecules. All standard molecules were solubilized in water–acetonitrile (50 : 50,v/v). Level 2 was used for an MS/MS spectral data match of a specific compound in electronic libraries such as the HMDB database, the METLIN database and the KEGG database, but with no standards available. Level 3 was used only for chemical class assignments where the exact identity of the compound could not be verified. To further illustrate whether the observed differences in the metabolites reflect coordinated alternations in defined metabolic pathways, a pathway analysis was performed in Metabo Analyst 3.0 (http://www.metaboanalyst.ca/MetaboAnalyst/).

### 2.9 Model construction

Based on the metabolomic change, we constructed a OPLS-DA model for anticancer mechanism prediction of candidates. Meanwhile, for the purpose of evaluating the robustness of the model, sevenfold cross validation was applied which was further confirmed by analysis of variance (CV-ANOVA). In addition, the model was likely to suffer from overfit even though the *Q*^2^ value satisfies the acceptable threshold, which prompted us to employ a permutation test for verifying the model given.

### 2.10 Evaluation and application of the model

To evaluate the accuracy of the above model, the model was validated with the metabolic profiling data of gemcitabine (GEM), carmustine (BCNU), mitoxantrone (MIT) and vindesine (VDS), and the four drugs belonging to the four categories previously used to construct the model. In the next step we used the model to predict the potential mechanism of apigenin (API) and diosgenin (DIO), which have been reported to have an unclear anticancer effect.

## 3. Results

### 3.1 Method validation

**Fig. 1:**
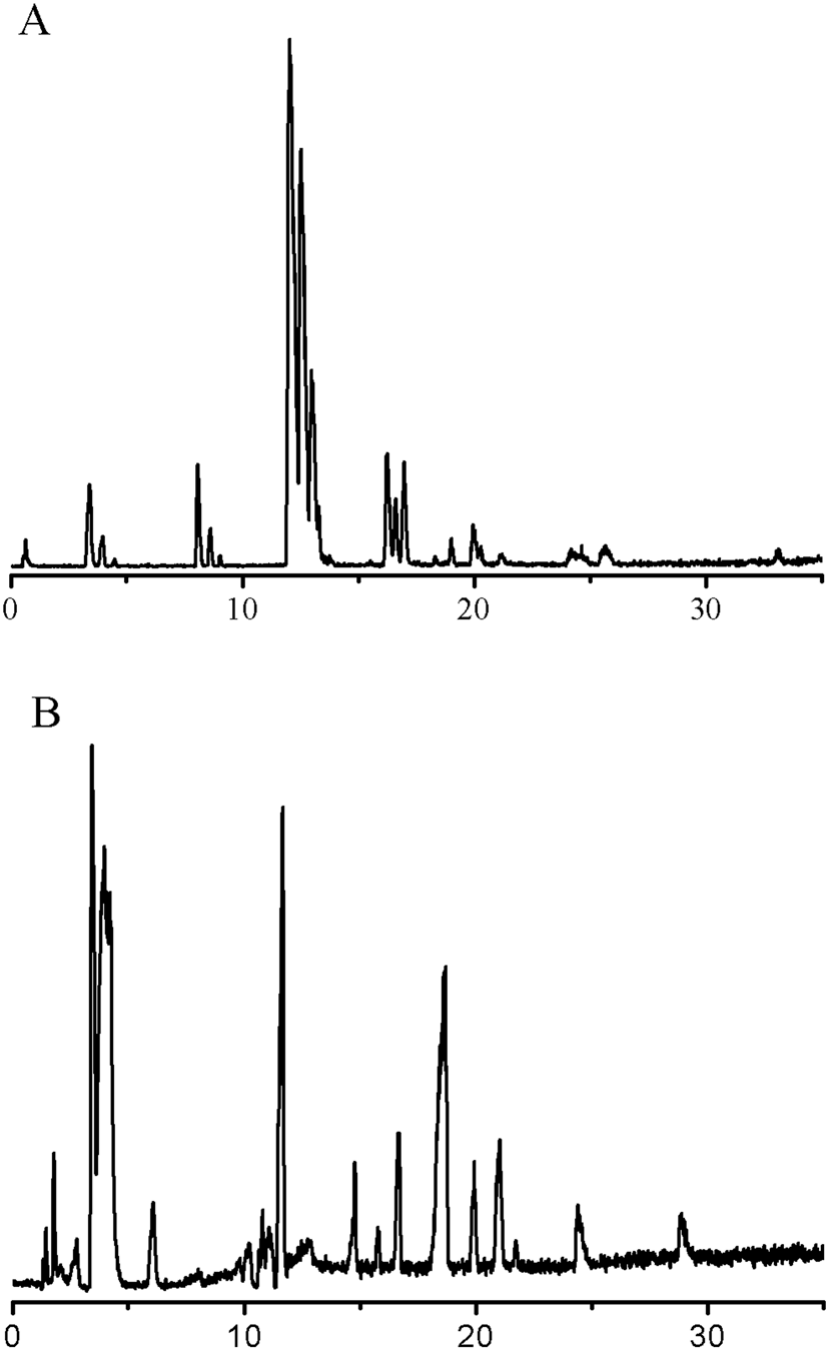
Typical total ion chromatograms of cellular metabolites. (A) RPLC method; (B) HILIC method.

Two typical total ion chromatograms obtained from the RPLC method and HILIC method are illustrated in Fig. 1. The repeatability results are shown in Table 1; the RSD of retention time for the RPLC and HILIC analyses ranged from 0.04% to 1.2% and 0.08% to 0.10% respectively, while the RSD of relative peak area ranged from 11% to 15% and 3.0% to 8.5% respectively, indicating the good reproducibility of this method. The RSDs of stability (<15%) showed that the preparation sample stored at room temperature was stable for 24 h. In addition, we imported the QC sample data into SIMCA13.0 for PCA analysis and all of the QC samples shifted a small extent within 2 SD, suggesting excellent systematic stability during the whole experiment (Fig. S7, ESI†). These quality assessments showed that the method was reliable and that any observed differences between the test samples would reflect variations in their metabolite profiles rather than technical errors.

**Table 1:** Repeatability in retention time and peak area of seven common peaks in blank cell extract obtained by RP-UPLC-MS (*n* = 6) and HILIC-UPLC-MS (*n* = 6)

Mode	Metabolite	*m*/*z*	RSD/RT (%)	RSD/area (%)
RPLC	Methyl malonyl carnitine	262.3071	1.2	14
RPLC	3-Methylglutaryl-carnitine	290.3483	0.14	14
RPLC	Hexadeca-sphinganine	274.3148	0.30	15
RPLC	Dihydro-sphingosine	302.3059	0.09	12
RPLC	Cyclic GMP	346.3753	0.18	11
RPLC	Testosterone propionate	345.2750	0.27	12
RPLC	DG(16 : 1(9Z)/20 : 1(11Z)/0 : 0)	621.4358	0.04	15
HILIC	l-Phenylalanine	165.9617	0.10	8.5
HILIC	l-Methionine sulfone	182.0782	0.09	4.7
HILIC	l-Glutamic acid	148.0601	0.09	3.0
HILIC	Glutamine	147.0724	0.08	7.8
HILIC	Glycerophosphocholine	258.1533	0.08	4.9
HILIC	4-Pyridoxic acid	184.1304	0.09	3.7
HILIC	Histidine	156.0900	0.08	7.0

### 3.2 Quality assessment of the LC-MS data

To illustrate the general trend, unsupervised PCA was performed, and the results of the PCA of the untargeted dataset are displayed in Fig. S8 (ESI†). The first two principal components accounted for only 47% of the overall variability. In the PCA score plot, each data point represented one sample, and the distance between points in the score plot was an indication of similarity between samples. The results indicated that the four groups clustered and separated respectively, which may be caused by the action of the four drugs on the twelve tumor cells. However, some sample points overlapped with the other group and any individual outlier was ruled out.

### 3.3 Selection and identification of differential metabolites

After OPLS-DA analysis, based on the VIP values (VIP > 1.0), a list of altered metabolites making the greatest contribution to the classification were screened. Among them, the concentrations of 11, 17, 12 and 12 metabolites for each drug-treated group (antimetabolic agents, agents directly acting on DNA, agents which affect microtubule dynamics, and RNA interference agents) were observed to be significantly different (*p* < 0.05) between the two groups.

**Fig. 2:**
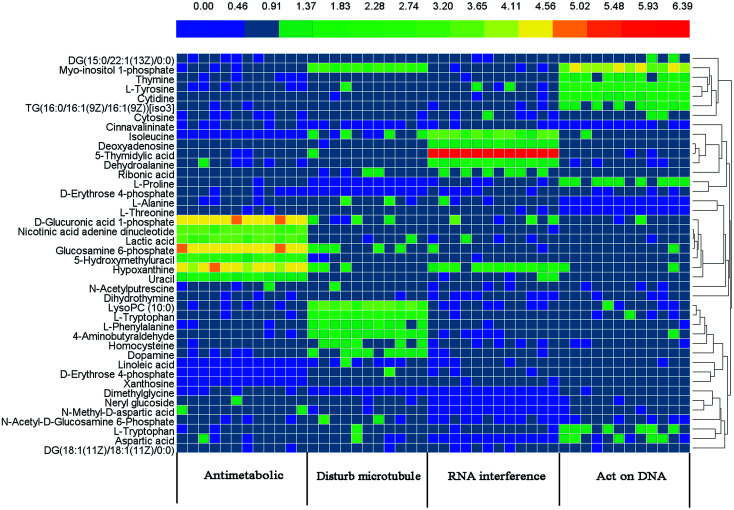
Clustered heat map of the metabolic profiles of four drug treated groups. Each row represents a different metabolite, and columns represent the samples from four drug treated groups. Different colors represent the concentration of different metabolites, where red represents a high concentration and blue represents a low concentration.

Tables 2–5 summarise the marker metabolite information and the fold change (FC), with a positive value indicating a relatively higher concentration is present in the drug-treated group, while a negative value suggests a relatively lower concentration is present in treatment group. To understand the difference between the metabolites across the four drug treated groups, the 42 metabolites were visualized in a clustered heat map (Fig. 2), with colors based on the actual FC values. The metabolites were classed into four clusters, which demonstrated the ability of metabolomics to distinguish the mechanism of action of different anticancer drugs.

**Table 2:** Significant altered metabolites induced by drugs that affect microtubule dynamics^a^

No.	RT/min	Biomarker	*m*/*z*	*P* value	AUC	FC	Trend	Metabolite pathway
1	35.704	DG(15 : 0/22 : 1(13Z)/0 : 0)	654.5905	**P < 0.01	0.674	1.298	↑	Glycerolipids metabolism
2	39.784	Myo-inositol 1-phosphate	261.0414	*P < 0.05	0.805	3.469	↑	Inositol phosphate metabolism
3	24.220	Cinnavalininate	301.0518	*P < 0.05	0.678	0.816	↓	Tryptophan catabolism
4	39.799	LysoPC (10 : 0)	413.3280	*P < 0.05	0.819	3.592	↑	Phospholipid metabolism
5	21.039	4-Aminobutyraldehyde	88.0743	*P < 0.05	0.546	1.628	↑	Arginine catabolism
6	11.865	Dimethylglycine	104.0729	*P < 0.05	0.561	0.620	↓	Glycine metabolism
7	15.049	l-Proline	116.0712	*P < 0.05	0.582	0.314	↓	Arginine and proline metabolism
8	15.609	Homocysteine	136.0404	**P < 0.01	0.655	1.632	↑	Cysteine and methionine metabolism
9	15.405	Dopamine	154.0883	*P < 0.05	0.606	1.440	↑	Tyrosine catabolism
10	13.764	l-Phenylalanine	165.9617	*P < 0.05	0.621	1.724	↑	Phenylalanine metabolism
11	12.077	L-Tryptophan	205.0541	*P < 0.05	0.547	2.195	↑	Tryptophan metabolite
12	17.052	d-Erythrose 4-phosphate	223.0174	**P < 0.01	0.501	0.644	↓	Pentose phosphate metabolism

a “↑” indicates that the metabolite content in the drug-treated group is higher than that in the control group; “↓” indicates that the metabolite content in the drug-treated group is lower than that in the control group; the *P* value was calculated using One Way ANOVA with the Bonferroni correction (significance at *P < 0.05, **P < 0.01).

**Table 3:** Significant altered metabolites induced by drugs that damage DNA structure^a^

No.	RT/min	Biomarker	*m*/*z*	*P* value	AUC	FC	Trend	Metabolite pathway
1	24.322	Cinnavalininate	301.2291	*P < 0.05	0.712	0.756	↓	Tryptophan catabolism
2	35.764	DG(15 : 0/22 : 1(13Z)/0 : 0)	654.5937	**P < 0.01	0.704	1.274	↑	Glycerolipids metabolism
3	39.875	TG(16 : 0/16 : 1(9Z)/16 : 1(9Z))[iso3]	820.7550	*P < 0.05	0.762	1.564	↑	Glycerolipids metabolism
4	39.780	Myo-inositol 1-phosphate	261.0370	*P < 0.05	0.804	4.177	↑	Inositol phosphate metabolism
5	40.722	DG(18 : 1(11Z)/18 : 1(11Z)/0 : 0)	638.5786	*P < 0.05	0.645	1.041	↑	Glycerolipids metabolism
6	20.912	4-Aminobutyraldehyde	88.0744	*P < 0.05	0.616	1.149	↑	Arginine catabolism
7	15.647	l-Alanine	90.0564	*P < 0.05	0.539	0.285	↓	Alanine metabolism
8	10.820	Cytosine	112.0494	*P < 0.05	0.524	1.224	↑	Pyrimidine metabolism
9	15.047	l-Proline	116.0708	*P < 0.05	0.671	1.372	↑	Arginine and proline metabolism
10	11.907	l-Threonine	120.0678	*P < 0.05	0.654	0.661	↓	l-Threonine metabolism
11	11.690	Thymine	127.0493	*P < 0.05	0.522	1.959	↑	Pyrimidine metabolism
12	20.908	Dihydrothymine	129.0671	**P < 0.01	0.558	0.965	↓	Pyrimidine metabolism
13	16.862	*N*-Acetylputrescine	131.1208	*P < 0.05	0.651	0.968	↓	Arginine catabolism
14	15.421	Dopamine	154.0849	*P < 0.05	0.575	1.166	↑	Tyrosine catabolism
15	15.584	l-Tyrosine	181.9096	*P < 0.05	0.609	2.705	↑	Tyrosine metabolism
16	12.031	L-Tryptophan	205.0562	*P < 0.05	0.533	1.603	↑	Tryptophan metabolite
17	15.068	Cytidine	244.0982	**P < 0.01	0.597	1.835	↑	Pyrimidine metabolism

a “↑” indicates that the metabolite content in the drug-treated group is higher than that in the control group; “↓” indicates that the metabolite content in the drug-treated group is lower than that in the control group; the *P* value was calculated using One Way ANOVA with the Bonferroni correction (significance at *P < 0.05, **P < 0.01).

**Table 4:** Significant altered metabolites induced by antimetabolic drugs^a^

No.	RT/min	Biomarker	*m*/*z*	*P* value	AUC	FC	Trend	Metabolite pathway
1	12.260	d-Glucuronic acid 1-phosphate	275.0116	***P < 0.001	0.696	4.932	↑	Amino sugar and nucleotide sugar metabolism
2	16.821	Uracil	113.0351	**P < 0.01	0.783	1.808	↑	Pyrimidine metabolism
3	0.661	Hypoxanthine	136.9225	***P < 0.001	0.652	4.736	↑	Pyrimidine metabolism
4	40.628	Nicotinic acid adenine dinucleotide	666.1287	*P < 0.05	0.722	3.708	↑	Act as second messenger
5	33.008	Glucosamine 6-phosphate	282.2731	*P < 0.05	0.643	4.980	↑	Alanine, aspartate and glutamate metabolism
6	39.893	Linoleic acid	303.3405	*P < 0.05	0.522	0.166	↓	Lipid metabolism
7	13.055	Isoleucine	131.9333	***P < 0.001	0.678	0.013	↓	Valine, leucine and isoleucine biosynthesis
8	36.194	5-Hydroxymethyluracil	143.0419	***P < 0.001	0.650	3.027	↑	—
9	11.862	Lactic acid	91.0408	***P < 0.001	0.543	3.800	↑	Glycolysis or gluconeogenesis
10	1.879	d-Erythrose 4-phosphate	218.0164	***P < 0.001	0.609	0.019	↓	Pentose phosphate pathway
11	33.832	Xanthosine	307.0836	*P < 0.05	0.543	0.169	↓	Purine metabolism

a “↑” indicates that the metabolite content in the drug-treated group is higher than that in the control group; “↓” indicates that the metabolite content in the drug-treated group is lower than that in the control group; the *P* value was calculated using One Way ANOVA with Bonferroni correction (significance at *P < 0.05, **P < 0.01, ***P < 0.001); “—” means that there is no corresponding metabolite pathway determined by MetaboAnalyst 3.0.

**Table 5:** Significant altered metabolites induced by drugs that interfere with RNA^a^

No.	RT/min	Biomarker	*m*/*z*	*P* value	AUC	FC	Trend	Metabolite pathway
1	39.952	Ribonic acid	167.0589	***P < 0.001	0.520	1.562	↑	—
2	12.269	Deoxyadenosine	274.1097	***P < 0.001	0.875	1.646	↑	Purine metabolism
3	12.759	5-Thymidylic acid	340.2486	**P < 0.01	0.843	6.795	↑	Pyrimidine metabolism
4	40.002	Neryl glucoside	345.0645	**P < 0.01	0.807	0.004	↓	—
5	16.917	Uracil	113.0375	**P < 0.01	0.521	1.011	↑	Pyrimidine metabolism
6	0.675	Hypoxanthine	136.9216	***P < 0.001	0.821	2.487	↑	Pyrimidine metabolism
7	12.983	Isoleucine	131.9327	***P < 0.001	0.912	3.769	↑	Valine, leucine and isoleucine biosynthesis
8	19.790	Aspartic acid	134.0478	***P < 0.001	0.615	0.411	↓	Alanine, aspartate and glutamate metabolism
9	19.792	Dehydroalanine	88.0386	***P < 0.001	0.812	1.856	↑	Methylation metabolism
10	11.429	Dimethylglycine	104.0691	**P < 0.01	0.512	0.136	↓	Methylation metabolism
11	15.802	*N*-Methyl-d-aspartic acid	148.0639	**P < 0.01	0.735	0.021	↓	—
12	1.795	*N*-Acetyl-d-glucosamine 6-phosphate	319.0641	***P < 0.001	0.631	0.106	↓	Amino sugar and nucleotide sugar metabolism

a “↑” indicates that the metabolite content in the drug-treated group is higher than that in the control group; “↓” indicates that the metabolite content in the drug-treated group is lower than that in the control group; the *P* value was calculated using One Way ANOVA with the Bonferroni correction (significance at *P < 0.05, **P < 0.01, ***P < 0.001); “—” means that there is no corresponding metabolite pathway determined by MetaboAnalyst 3.0.

**Fig. 3:**
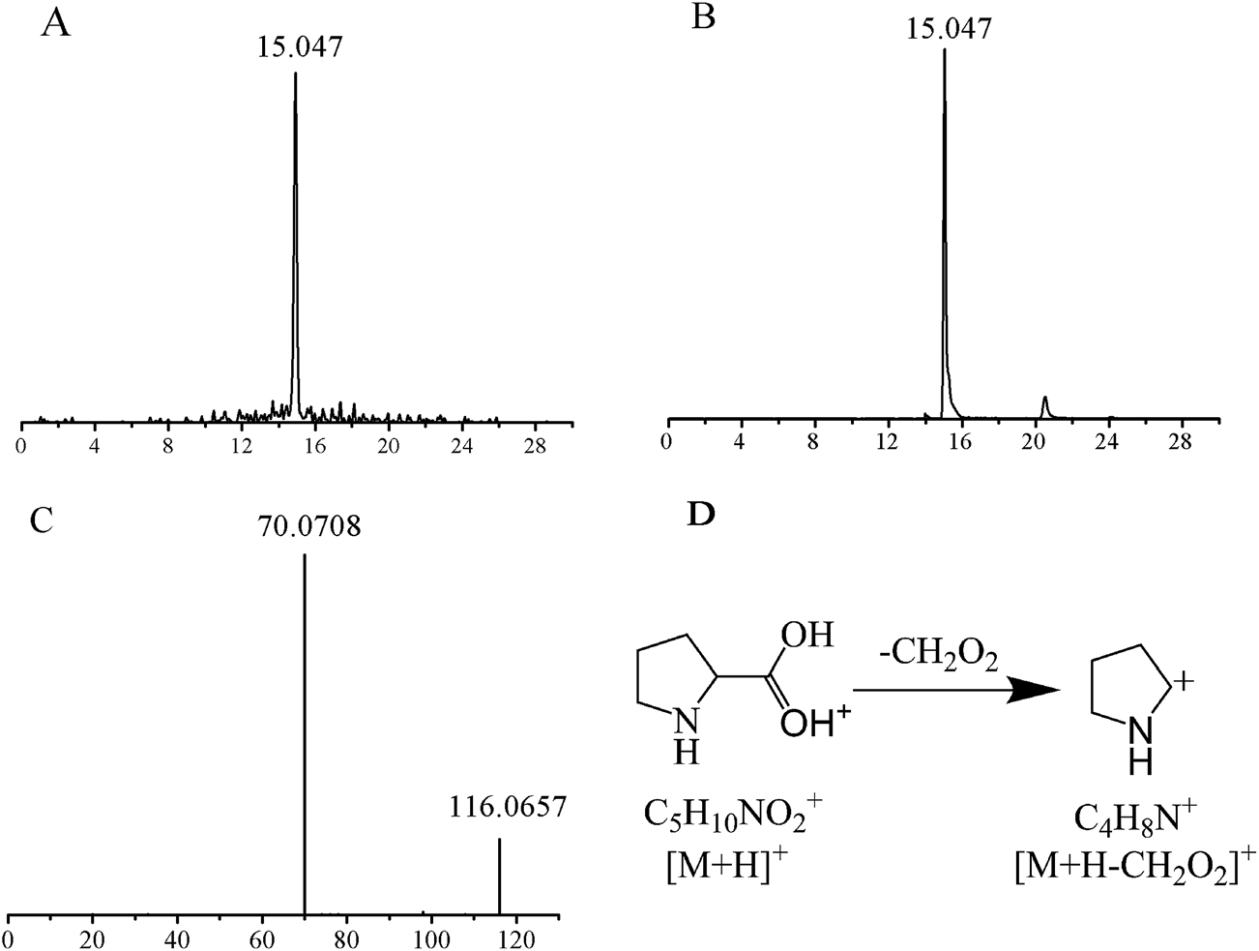
Identification of a selected biomarker candidate (*m*/*z* 116.0708). (A) Extracted ion chromatogram of *m*/*z* 116.0708; (B) total ion chromatogram of a commercial l-proline standard (molecular weight = 115.1305); (C) LC-MS/MS spectrum of the *m*/*z* 116.0708 ion in the positive ion mode; (D) fragmentation pathways of l-proline.

The structures of these candidates were identified using the method described above, by which 9 were identified using standard molecules, 30 were identified by matching to a database, and the rest by fragmentation pathway (shown in Table 2). For example, the identification of the candidate at *m*/*z* 116.0708 (retention time: 15.047 min) is shown in Fig. 3. First, the accordant quasimolecular ion peak was determined based on its retention time in Masslynx with *m*/*z* 116.0708 (Fig. 3A). Then the candidate was found to be l-proline by searching the HMDB and METLIN databases. Finally, the result was confirmed by comparison with the retention time (Fig. 3B) and the MS/MS spectrum of an l-proline standard (Fig. 3C). A fragmentation pathway was used to identify other metabolites with similar structures.

### 3.4 Construction and validation of metabolite-based prediction model

**Fig. 4:**
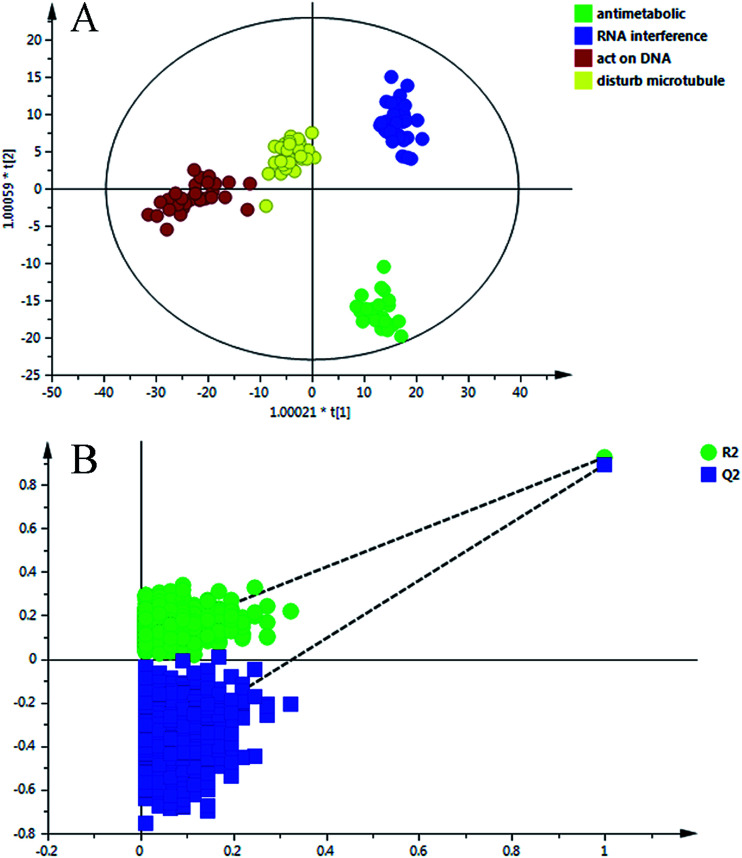
(A) OPLS-DA scores scatter plot of the four classes on the RP + HI dataset; (B) permutation test of the OPLS-DA model.

The data matrix (retention time–mass pairs, sample names and normalized peak areas) obtained by RPLC and HILIC was utilized to construct OPLS-DA models respectively. Then the data were combined together, namely RP + HI, and the quality of the model was evaluated using a number of evaluation values such as the parameter *R*^2^, which indicated the goodness of fit, and *Q*^2^, which represented the model’s predictability. It can be seen from Table S3 (ESI†) that after data integration the values of *R*^2^ (cum.) and *Q*^2^ (cum.) were 0.909 and 0.869, which were larger than the parameters of the samples analyzed in one analysis mode. The results highlighted the advantages of the method using the RPLC/HILIC method for the analysis of both polar and non-polar metabolites within a complex cell sample. Once the data structure had been chosen, the OPLS-DA prediction model (Fig. 4A) was constructed with all of the samples from the four categories.

To validate the stability and accuracy of the model, sevenfold cross validation was applied which was further confirmed by the analysis of variance CV-ANOVA with a *p* value ≪0.01, which is shown in Table S4 (ESI†). Of the 999 random permutations, all *R*^2^ (cum.) and *Q*^2^ (cum.) values computed from the permuted dataset were below those from the original dataset, and the intercept values of *R*^2^ = 0.0871 and *Q*^2^ = −0.4360 are demonstrated in Fig. 4B. Overall, these results indicated that the predictive capabilities of the OPLS-DA models of the LC-MS data were reliable.

### 3.5 Evaluation of prediction models with new sample sets

As shown in Fig. 4, the new data dots of different drugs are located in different regions. The four drugs were considered: disturbing microtubule dynamics (VDS), embedding between a double-stranded DNA (MIT), alkylating nucleic acid chain (BCNU) and cytidine derivatives (GEM). The dots of VDS completely overlapped with those of agents that affect microtubule dynamics, with *R*^2^ = 0.718 and *Q*^2^ = 0.674. The process was replicated, and a lot of sample points of GEM, BCNU and MIT were distributed in the corresponding area. In general, these results were in agreement with the actual antitumor mechanism. These results may partially prove that the model could accurately predict the anticancer mechanism of candidates that belonged to the four categories.

### 3.6 Application of the prediction model

**Fig. 5:**
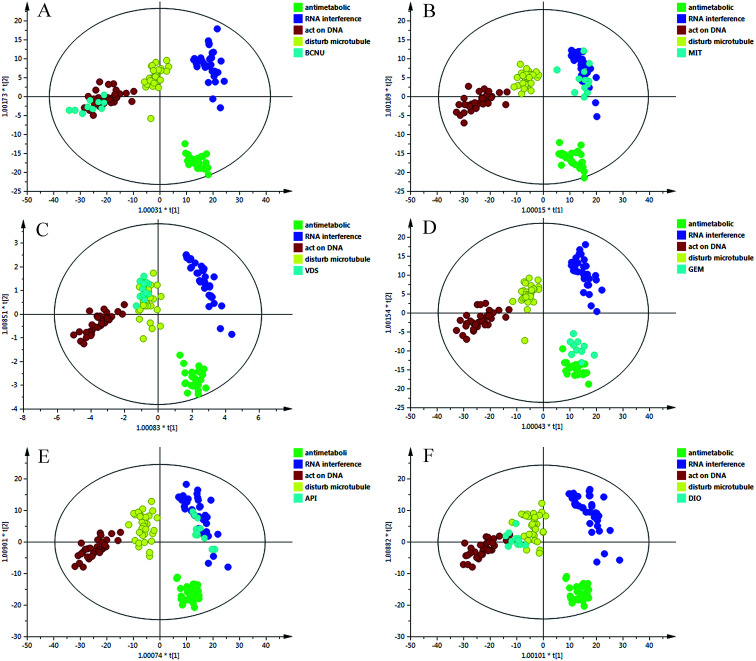
The OPLS-DA score plots of the different treated groups and four classes. (A) BCNU; (B) MIT; (C) VDS; (D) GEM; (E) API; (F) DIO.

To further evaluate the performance of the model in mechanism prediction, the cell lines were treated with API and DIO in the same method described above. Fig. 5 shows that most of the data points which represent the API treatment group are located in the same area as those for agents that affect microtubule dynamics, with *R*^2^ = 0.838 and *Q*^2^ = 0.762, suggesting that the API was likely to have an anticancer effect by interfering with microtubules. However, the data dots of DIO fell in the region between those of agents that affect microtubule dynamics and those of agents that directly act on DNA. The parameters of *R*^2^ = 0.618 and *Q*^2^ = 0.589 indicated that the prediction model was feasible but not very accurate when it was applied to DIO. This explained and highlighted that DIO may have a complicated anticancer effect on tumor cells.

## 4. Discussion

### 4.1 Optimization of analysis method

Our study sought to analyze a great deal of metabolites to gather real-time cell metabolomic information and thus deepen the knowledge of drug effects on cancer cells. Considering that direct methanolic extraction was also preferred in many biological samples, the extraction solution was optimized according to the peak number in the TIC, which is shown in Fig. 1. At first, a liquid–liquid extraction method was applied with chloroform–methanol–water (4 : 4 : 2,v/v/v). However, there was a protein membrane between the upper and lower layers of the two-phases, which caused trouble in the sampling process and introduced a large deviation to the subsequent analysis. Thus a binary solvent (methylene chloride and methanol) was utilized, which better facilitated sample handling and substituted dichloromethane for chloroform, thereby reducing harm to the operator.

### 4.2 Effects of anticancer agents on metabolic profiles

**Fig. 6:**
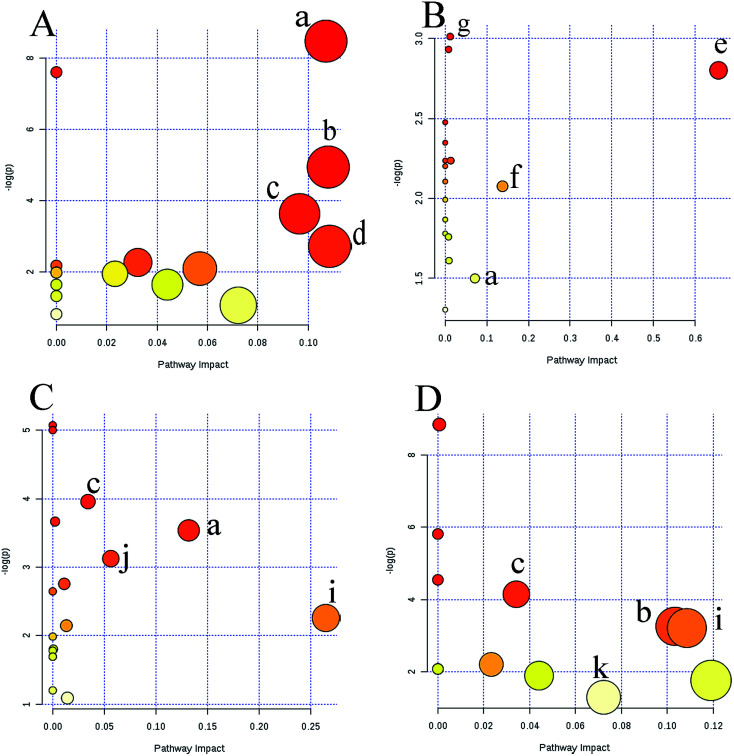
Overview of pathway analysis affected by four groups based on MetaboAnalyst 3.0. (A) agents directly acting on DNA; (B) antimetabolic agents; (C) RNA interference agents; (D) agents that affect microtubule dynamics. (a) Pyrimidine metabolism; (b) arginine and proline metabolism; (c) glycine, serine and threonine metabolism; (d) tryptophan metabolism; (e) linoleic acid metabolism; (f) pyruvate metabolism; (g) amino sugar and nucleotide sugar metabolism; (h) purine metabolism; (i) alanine, aspartate and glutamate metabolism; (j) aminoacyl-tRNA biosynthesis; (k) tyrosine metabolism; (l) tryptophan metabolism; (m) phenylalanine metabolism.

Metabolic profiling not only reveals the individual metabolite alteration but also provides an integrated view of the metabolic processes induced by anticancer candidates. With multivariate and univariate statistical analyses, 42 metabolites were tentatively identified and an OPLS-DA model was constructed to preliminarily predict the possible mechanism of candidates. Based on the metabolite information, a map of anticancer drug related metabolic pathways was constructed and is shown in Fig. 6. Several metabolic pathways were involved in the modifications between the groups. MetaboAnalyst was used to assign different pathways for the feature compounds.

The dots in Fig. 6 represent the pathways that were matched using pathway impact values from pathway topology analysis and *p* values from pathway enrichment analysis. Pyrimidine metabolism, purine metabolism, arginine and proline metabolism, glycine, serine and threonine metabolism, tryptophan metabolism, linoleic acid metabolism, pyruvate metabolism, amino sugar and nucleotide sugar metabolism, alanine, aspartate and glutamate metabolism, phenylalanine metabolism, tyrosine metabolism, and aminoacyl-tRNA biosynthesis, were selected as the most important altered metabolic pathways. These affected pathways were highly related to amino acid metabolism, nucleotide metabolism and lipid metabolism. These intrinsic metabolism disorders may highly relate to their principal metabolic pathways, and maintain unlimited proliferation and thus are vulnerable to anticancer drugs that impair their vital metabolic pathways.^21^

### 4.3 The potential anticancer mechanism of API and DIO

Apigenin (5,7,4′-trihydroxyflavone) is a flavonoid compound, and it has been reported that apigenin inhibits tumor cell proliferation, induces tumor cell apoptosis and could inhibit tumor angiogenesis, invasion and metastasis, and also interferes with the signal transduction pathway and anti-oxidation of tumor cells.^22^ The metabolic profiling model was applied for the first time to explore metabolic effects of apigenin in tumor cell lines. The method predicted that API may have a similar anticancer mechanism to agents that affect microtubule dynamics. In terms of recent research on mechanisms of API, API may exert its anticancer activity by interfering with microtubule dynamics through multiple molecular pathways.

First, Wen investigated that after treatment with apigenin, the amount of tubulin was reduced and was irregularly arranged in HepG2 cells.^23^ This phenomenon may be caused by the perturbations in microtubule dynamics, because apigenin bounded tubulin, inhibited tubulin polymerization into microtubule, distorted microtubule structure and caused apoptosis.^24^ In addition, some molecular interactions studies concerning apigenin influence upon microtubules have been reported, such as up-regulated p21/WAF1 expression,^25^ inhibition of the expression of cyclinD1 and phosphorylation of Rb protein,^26^ and controlling the expression of vascular endothelial growth factor (VEGF) and hypoxia-inducible factor 1 alpha (HIF-1alpha).^27^ Thus the prediction results gave a reliable direction of mechanisms and were consistent with all the research mentioned above.

Diosgenin is a naturally occurring steroidal sapogenin, and it has been reported that diosgenin affects various phases of tumorigenesis including tumor cell proliferation, cell migration, angiogenesis and apoptosis.^28^ The data dots of DIO were distributed in the region between those of agents that affect microtubule dynamics and those of agents that directly act on DNA, indicating that DIO was likely to have two anti-tumor mechanisms. On the one hand, it was proven by binding DNA, activating IjBa kinase and p65 phosphorylation, and inhibiting Akt activation, that DIO could suppress TNF-induced NF-jB activation,^29^ which is a requirement of cell life processes from the G phase to the S phase.^30^ Besides, treatment of HepG2 cells with diosgenin resulted in activating cpoly-ADP-ribose polymerase (PARP) and releasing cytochrome that is thought to be one of the early cell signals of DNA damage.^31^ Also, diosgenin strongly generated ROS and this oxidative stress might induce DNA damage.^32^ On the other hand, Akinori demonstrated that DIO led to a decrease of the α-tubulin and c-fos mRNA expression relating to the cell cycle.^33^ However, further study is required on the effects of DIO on microtubules in detail. The results of several other studies have shown that DIO may increase sustained phosphorylation of JNK, p38 MAPK and apoptosis signal-regulating kinase (ASK)-1, indicating that DIO may have a variety of anticancer mechanisms.^34^ The results presented here suggested that the constructed model might be able to achieve a better prediction of anticancer candidates with multiple mechanisms of action.

### 4.4 Advantages and directions

To our best of our knowledge, this is the first metabolic profile study of tumor cells exploring the possibility to construct a preliminary model for the research of anticancer mechanisms. In fact, most anticancer drugs are designed to decrease tumor cell proliferation and some anticancer drugs with different mechanisms may cause the same changes in nucleotide content. As depicted in the previous model, the two data points of antimetabolic agents and agents that directly act on DNA were overlapped partially, which may be caused by the fact that antimetabolic agents not only competitively antagonize endogenous nucleotides but also indirectly perturb the pathway of DNA synthesis. In addition, the parameters of the previous model were *R*^2^ = 0.744 and *Q*^2^ = 0.665, demonstrating that this prediction model was practical and predictive to some extent. Compared with the previous study, our present study has provided integrative insights on metabolic shifts during drug action, and the new method can yield more new biomarkers to help reveal the mechanism of anticancer drug action. The significantly perturbed metabolic pathways included energy metabolism, amino acid metabolism and lipid metabolism, which showed that the constructed model is highly related to drug action. In this new model, four groups cluster and separate respectively with *R*^2^ = 0.909 and *Q*^2^ = 0.869, and this meant that the present method was more accurate and stable than the previous one. Furthermore, the use of the RPLC/HILIC combined method for the analytes provided a comprehensive metabolite profile on the cell changes.

However, our study needs further improvements. First, given that the status of adherent tumor cells was more stable than that of suspension cells, we only chose twelve typical adherent tumor cells for this study, which belonged to different tissues cells or the same cancer transferred to different organs such as DU145 and PC3. To expand the application and improve the accuracy of the method, more cell lines and more drugs should be involved. Second, using different analysis platforms together can validate and complement each other, which can provide more information on the connection between metabolites and drug actions. Third, it will be helpful to provide metabolite concentrations to support this hypothesis, not only based on the literature but also experimental data. Extra work, *e.g.*, an assay panel and quantitative study should be done for the confirmation of the method. Certainly, the new method in this paper can predict the mechanism of action of anticancer candidates early. But for a better understanding of how compounds or drug candidates interact with wide-ranging molecular targets and the biochemical consequences of these interactions, traditional strategies for mechanism research still need to be performed, such as Flow Cytometry (FCM), Polymerase Chain Reaction (PCR), western Blotting (WB), and so on.

## 5. Conclusions

In conclusion, by using LC-MS based cell metabolic profiling coupled with multivariate and univariate statistical analyses, a large overview of metabolic shifts in treated groups have been presented. These 42 metabolites might be tied to several important metabolic pathways, which might be potential targets for future research on anticancer candidate mechanisms. Based on these metabolites, the first OPLS-DA prediction model has been successfully constructed for investigating the mechanism of anticancer candidates. Additionally, the results showed that the prediction model performed well when it was validated, and the results were similar to the actual anti-tumor mechanism. Finally, two natural compounds were used and the prediction model proven to be robust and accurate against biases caused by different mechanisms of action, which validated that the model was also very useful for the prediction of anticancer candidates with multiple mechanisms. Thus, our work is the first study providing a reliable rapid screening method to evaluate the preliminary efficacy of anticancer compounds on tumour cells that would hopefully facilitate the development of safer drugs and help improve the development efficiency.

## Compliance with ethical requirements

The study has been approved by the ethics committee of the medical faculty at the Shenyang Pharmaceutical University.

## Conflicts of interest

The authors declare that they have no conflict of interest.

## Supplementary Material

RA-008-C8RA00242H-s001
