# Supplementary material for: LC-MS based cell metabolic profiling of tumor cells: a new predictive method for research on the mechanism of action of anticancer candidates
Source: RSC Adv. 2018 May 8;8(30):16645–56. doi: 10.1039/c8ra00242h (PMC9080298; doi:10.1039/c8ra00242h)
Supplement: RA-008-C8RA00242H-s001 [file RA-008-C8RA00242H-s001.pdf]

Electronic Supplementary Material (ESI) for RSC Advances.
This journal is © The Royal Society of Chemistry 2018

Supplementary Materials

Table 3 R^2 and Q^2 intercept values for the OPLS-DA mode

Name	R^2	Q^2
RP	0.640	0.604
HI	0.752	0.669
RP+HI	0.909	0.869
VDS	0.718	0.674
GEM	0.791	0.715
BCNU	0.682	0.638
MIT	0.819	0.727
API	0.838	0.762
DIO	0.618	0.589

Note: RP are the database from RPLC method, HI are the database from HILIC method, RP+HI means that the RP and HI data was combined together.

Table 4 CV-ANOVA parameters derived from cross-validation of OPLS-DA model

	SS	df	MS	F	*P*	SD
**Total corr.**	360	360	1			1
**Regression**	239.189	42	5.695	14.990	4.01E-20	2.386
**Residual**	120.811	318	0.380			0.616

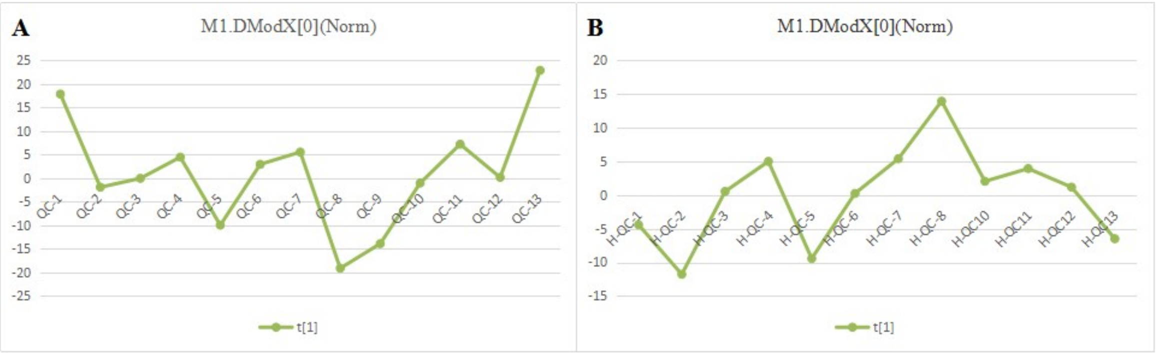

Fig.7 Stability of UPLC-MS sysytem in ananlysis

A:RPLCmethod; B:HILIC method

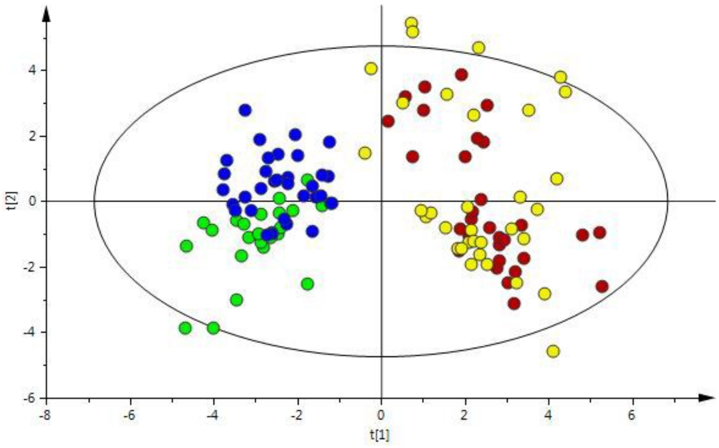

Fig.8 The PCA score plot of cells in drug-treated groups of four classes

R^2= 0.426 Q^2= 0.147
